# The association between academic emotions and self-efficacy among nursing undergraduates: a cross-sectional regression analysis

**DOI:** 10.3389/fpsyg.2026.1901679

**Published:** 2026-07-24

**Authors:** Lili Guo, Danfeng Yan, Zhi Chai, Baotian Zhang, Meng Guo, Zhe Zhang, Rong Wu, Yukai Feng, Rui Jiao

**Affiliations:** 1 School of Nursing, Shanxi University of Chinese Medicine, Jinzhong, China; 2 Shanxi Mental Health Center, Taiyuan Psychiatric Hospital, Taiyuan Fifth People's Hospital, School of Mental Health, Shanxi Medical University, Mental Health Hospital of Shanxi Medical University, Taiyuan, China

**Keywords:** academic emotions, multiple linear regression, nursing undergraduates, predictors, self-efficacy

## Abstract

**Background:**

Based on the Control-Value Theory of Achievement Emotions, this study aimed to examine the associations between academic emotions and self-efficacy among nursing undergraduates, and to provide a theoretical basis for optimizing nursing education interventions.

**Methods:**

A cross-sectional study was conducted among 659 nursing undergraduates from a university. Data were collected using the Academic Emotions Scale and the Self-efficacy Scale. Statistical analyses were performed using SPSS, including descriptive statistics, correlation analysis, and multiple linear regression. The effects of different dimensions of academic emotions on self-efficacy were examined, while controlling for demographic variables such as gender and age.

**Results:**

The mean self-efficacy score of nursing undergraduates was 30.41 ± 5.15. Correlation analysis showed that confidence, excitement, and satisfaction were significantly positively correlated with self-efficacy (*r* = 0.56–0.67, *P* < 0.01), whereas anxiety, boredom, and frustration were significantly negatively correlated with self-efficacy (*r* = −0.16 to −0.23, *P* < 0.01). Multiple linear regression analysis indicated that, after adjustment for demographic variables, the model was statistically significant (*F* = 83.509, *P* < 0.001), explaining 50.7% of the variance in self-efficacy (*R*^2^ = 0.507, adjusted *R*^2^ = 0.501). Confidence and satisfaction were independent positive predictors of self-efficacy, whereas anxiety was an independent negative predictor. Although boredom showed a small positive association after adjustment, excitement and frustration were not independently associated with self-efficacy. No evidence of multicollinearity was observed.

**Conclusions:**

Academic emotions were significantly associated with self-efficacy among nursing undergraduates. Confidence and satisfaction were independent positive predictors of self-efficacy, whereas anxiety was the only independent negative predictor. Interventions aimed at fostering positive academic emotions and reducing anxiety may help enhance self-efficacy in nursing students.

## Introduction

With the continuous development of nursing education, improving the learning quality and professional competence of nursing undergraduates has become an important research focus. Self-efficacy, defined as an individual's belief in their ability to successfully perform specific tasks, is considered a key psychological factor influencing learning behaviors and academic performance (Artino, 2012; Bandura, 1977). According to Self-Efficacy Theory, self-efficacy not only affects learning motivation but also plays a critical role in academic persistence and learning engagement (Wu et al., 2020; Zarrin et al., 2023). Meanwhile, academic emotions have been reported to be associated with learning motivation and cognitive processing (Rentzios et al., 2025; Alshareef et al., 2024).

The Control-Value Theory of Achievement Emotions (CVT), proposed by Reinhard Pekrun in 2006, is a central theoretical framework in educational psychology for understanding achievement-related emotions (Pekrun, 2006). This theory posits that individuals' perceptions of control over learning activities and value attached to outcomes are the proximal cognitive antecedents of achievement emotions, which in turn exert profound influences on motivation, learning strategies, and academic achievement (Skues et al., 2025). According to CVT, academic emotions can be broadly categorized into positive emotions (e.g., enjoyment, pride) and negative emotions (e.g., anxiety, boredom), each showing distinct associations with learning behaviors. Academic emotions not only influence learning processes but are also closely associated with mental health. Positive emotions (e.g., confidence and satisfaction) are linked to higher learning engagement and better academic performance (Alshareef et al., 2024; Hall et al., 2016), whereas negative emotions (e.g., anxiety and boredom) may increase psychological stress and are associated with risks of anxiety and depression (Mesurado et al., 2018). In nursing education, this scale can help identify students at risk of adverse emotional states, evaluate the effectiveness of educational interventions, and inform psychological support and instructional strategies.

Previous studies have shown that positive academic emotions are associated with increased learning engagement and improved academic performance, whereas negative emotions have been associated with lower leaning motivation (López-Crespo et al., 2025; Tan et al., 2021). However, empirical evidence regarding the relationship between academic emotions and self-efficacy among nursing undergraduates remains limited. In particular, the independent effects of different dimensions of academic emotions on self-efficacy are not yet fully understood.

Therefore, this study aimed to examine the associations between different dimensions of academic emotions and self- efficacy among nursing undergraduates, in order to provide a theoretical basis for developing targeted educational interventions in nursing education.

## Methods

### Participants

A convenience sampling method was used to recruit 659 nursing undergraduates from a university. The inclusion criteria were: (1) full-time nursing undergraduate students; and (2) voluntary participation with written informed consent. Participants with incomplete questionnaires or responses containing obvious logical inconsistencies were excluded. The medical ethics committee of The Shanxi University of Chinese Medicine approved this study, and Ethics approval number is 2025LL171, as this study was not a clinical trial. The study was conducted in accordance with the Declaration of Helsinki. All participants provided informed consent before participation.

## Instruments

### Academic emotions scale

Academic emotions were assessed using a revised six-dimensional Academic Emotions Scale based on the Achievement Emotions Questionnaire (AEQ) developed by Pekrun (2006) and grounded in the control-value theory of achievement emotions (Fierro-Suero et al., 2020). The scale consists of six dimensions, including three positive emotions (confidence, excitement, and satisfaction) and three negative emotions (anxiety, boredom, and frustration). The total number of items is 24. All items are rated on a 5-point Likert scale ranging from 1 (strongly disagree) to 5 (strongly agree). Scores for each dimension are calculated by summing or averaging item scores. Higher scores indicate stronger intensity of the corresponding academic emotion. The scale has been widely applied in Chinese undergraduate populations and demonstrates good psychometric properties. The scale demonstrated good internal consistency (Cronbach's α = 0.863) and satisfactory construct validity, making it suitable for assessing academic emotions among university students.

### Self-efficacy scale

Self-efficacy was measured using the General Self-Efficacy Scale (GSES), which is grounded in the self-efficacy theory proposed by Bandura (1977, 2001). This scale is widely used to assess individuals' confidence in their ability to cope with various challenges. The GSES consists of 10 items that evaluate individuals' beliefs and coping abilities when facing difficulties and challenges. Each item is rated on a 4-point Likert scale, with higher scores indicating higher levels of self-efficacy. The scale has demonstrated excellent psychometric properties, including high internal consistency (Cronbach's α = 0.960), stable structure, and strong cross-cultural applicability. Self-efficacy is a key factor influencing learning behaviors and mental health; higher self-efficacy is associated with greater motivation and stronger problem-solving abilities (Bahari, 2025), whereas lower self-efficacy is linked to avoidance behaviors and learning burnout.

### Data collection

A cross-sectional survey design was employed, and data were collected through an online questionnaire. The questionnaire was developed using an online survey platform (Wenjuanxing) and distributed via class-based communication groups. Prior to data collection, participants were informed about the study purpose, procedures, and instructions. The survey was conducted anonymously, and standardized instructions were provided. Participants completed the questionnaire independently after providing informed consent. All data were used solely for research purposes. The questionnaire included three sections: demographic information, the Academic Emotions Scale, and the Self-efficacy Scale. To ensure data quality, all items were set as mandatory to prevent missing responses. Logical checks and response time thresholds were applied to identify and exclude invalid questionnaires. After excluding responses with excessively short completion times, patterned answers, or logical inconsistencies, a total of 659 valid questionnaires were retained, yielding an effective response rate of 83.6%. All data were exported, coded, and organized into a database for statistical analysis.

### Statistical analysis

All statistical analyses were conducted using SPSS (IBM Corp., Armonk, NY, USA) Statistics for Windows version 26.0. Continuous variables were tested for normality. Data with a normal distribution were expressed as mean ± standard deviation (mean ± SD), whereas non-normally distributed data were presented as median (interquartile range). Categorical variables were described using frequencies and percentages. Pearson correlation analysis was performed to examine the relationships between dimensions of academic emotions and self-efficacy. Multiple linear regression analysis was conducted with self-efficacy as the dependent variable and the six dimensions of academic emotions (confidence, excitement, satisfaction, anxiety, boredom, and frustration) as independent variables. Standardized regression coefficients (β), coefficients of determination (*R*^2^), and significance levels (*P* values) were reported. Multicollinearity was assessed using variance inflation factors (VIF). Demographic variables, including gender and age, were included in the regression model as covariates. To assess potential common method bias, Harman's single-factor test was conducted using exploratory factor analysis.

## Results

### Descriptive statistics

A total of 659 undergraduate nursing students participated in this study. The demographic characteristics of the participants are presented in Table 1. The mean age of the participants was 19.17 ± 1.33 years, and 77.8% were female. The descriptive statistics of academic emotions are presented in Table 2. Among the six dimensions, satisfaction had the highest mean score (4.05 ± 0.68), whereas boredom showed a relatively lower mean score (3.04 ± 0.92). The mean total score of self-efficacy was 30.41 ± 5.15.

**Table 1:** Demographic characteristics of the participants (*N* = 659).

Variable	n (%)/Mean ±SD
Age (years)	19.17 ± 1.33
Gender	
Male	146 (22.2%)
Female	513 (77.8%)
Grade	
First year	385 (58.4%)
Second year	106 (16.1%)
Third year	130 (19.7%)
Fourth year	38 (5.8%)
Place of origin	
Urban	332 (50.4%)
Rural	327 (49.6%)
Parental marital status	
Married	589 (89.4%)
Divorced	47 (7.1%)
Separated	23 (3.5%)

**Table 2:** Descriptive statistics of academic emotions among nursing undergraduates.

Variable	*N*	Mean	SD	Min	Max
Confidence	659	3.83	0.76	2	5
Excitement	659	3.83	0.78	1	5
Satisfaction	659	4.05	0.68	1	5
Anxiety	659	3.75	0.87	1	5
Boredom	659	3.75	0.87	1	5
Frustration	659	3.25	0.87	1	5
Positive academic emotions	659	46.81	8.21	19	60
Negative academic emotions	659	40.13	9.25	12	60
Self-efficacy	659	30.41	5.15	11	40

### Correlations between academic emotions and self-efficacy

**Figure 1:**
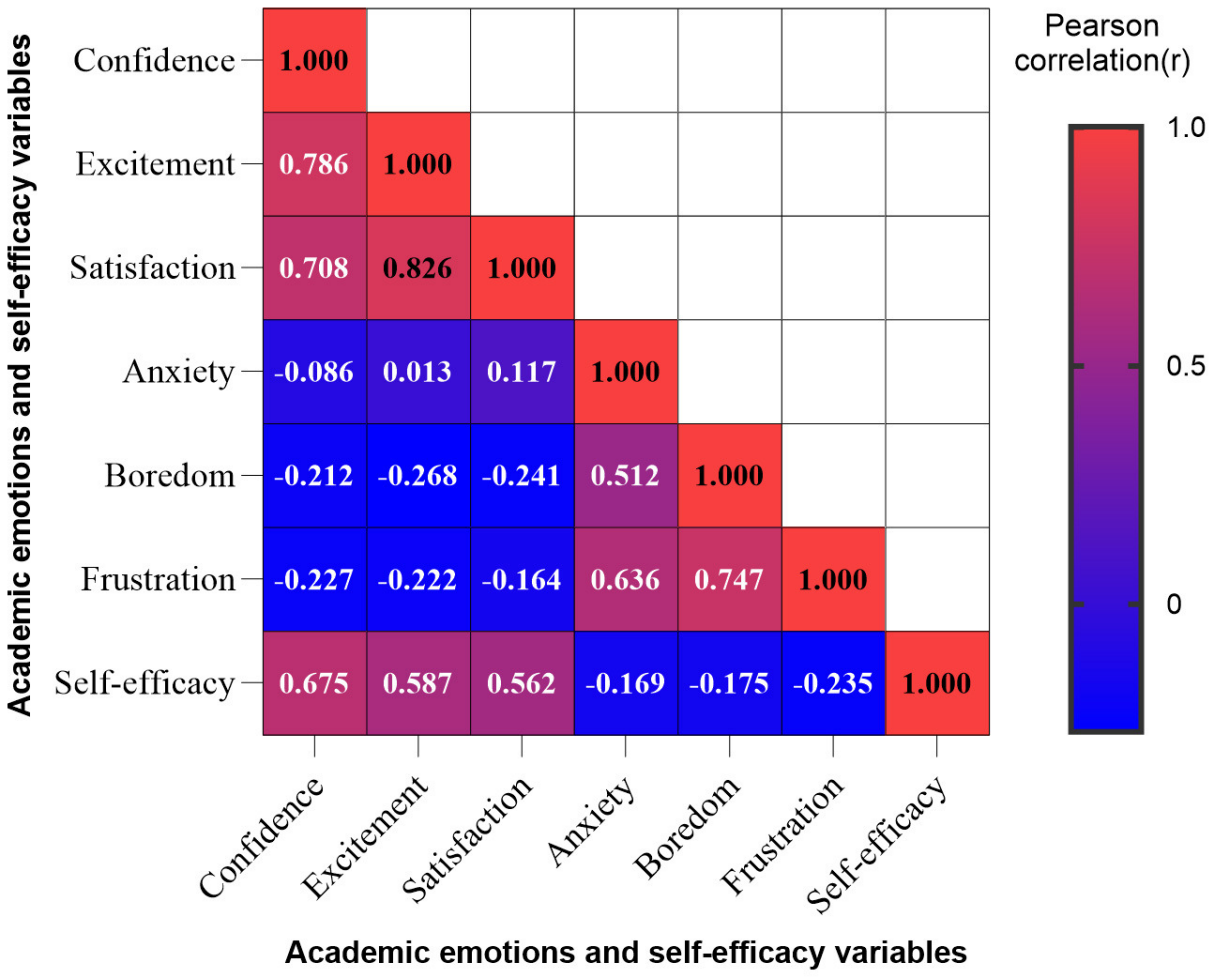
Correlation heatmap of academic emotions and self-efficacy among nursing students.

Pearson correlation analysis was conducted to examine the relationships between the dimensions of academic emotions and self-efficacy among nursing undergraduates. The results showed that confidence (*r* = 0.675, *P* < 0.001), excitement (*r* = 0.587, *P* < 0.001), and satisfaction (*r* = 0.562, *P* < 0.001) were significantly positively correlated with self-efficacy. In contrast, anxiety (*r* = −0.169, *P* < 0.001), boredom (*r* = −0.175, *P* < 0.001), and frustration (*r* = −0.235, *P* < 0.001) were significantly negatively correlated with self-efficacy. Among these, confidence showed the strongest positive correlation with self-efficacy, whereas frustration demonstrated a weak negative correlation, as shown in Figure 1.

Overall, academic emotions were significantly associated with self-efficacy among nursing undergraduates, with positive emotions showing positive correlations and negative emotions showing negative correlations.

### Multiple linear regression analysis

Multiple linear regression analysis was conducted with self-efficacy as the dependent variable, the six dimensions of academic emotions (confidence, excitement, satisfaction, anxiety, boredom, and frustration) as independent variables, and demographic variables (e.g., gender and age) as covariates. After controlling for demographic variables, the dimensions of academic emotions were entered into the regression model. Detailed coefficients for each predictor are presented in Table 3.

**Table 3:** Multiple linear regression analysis of academic emotion factors predicting self-efficacy (*N* = 659).

Predictor	*B*	SE	β	*t*	*P*	VIF
Constant	10.835	2.496	–	4.341	< 0.001	–
Gender	−0.879	0.350	−0.071	−2.512	0.012	1.054
Age	0.195	0.110	0.050	1.765	0.078	1.071
Confidence	3.107	0.318	0.459	9.755	< 0.001	2.913
Excitement	0.322	0.376	0.049	0.858	0.391	4.313
Satisfaction	1.751	0.389	0.233	4.496	< 0.001	3.532
Anxiety	−0.975	0.231	−0.165	−4.217	< 0.001	2.018
Boredom	0.604	0.246	0.108	2.459	0.014	2.531
Frustration	−0.305	0.276	−0.052	−1.103	0.271	2.897

*B*, unstandardized coefficient; SE, standard error; β, standardized coefficient; *t, t*-value; *P*, significance.

The results indicated that the overall model was statistically significant (*F* = 83.509, *P* < 0.001), explaining 50.7% of the variance in self-efficacy (*R*^2^ = 0.507, adjusted *R*^2^ = 0.501). Specifically, confidence (*B* = 3.107, β = 0.459, *P* < 0.001) and satisfaction (*B* = 1.751, β = 0.233, *P* < 0.001) were significant positive predictors of self-efficacy, indicating that higher levels of confidence and satisfaction were associated with higher self-efficacy. In contrast, anxiety (*B* = −0.975, β = −0.165, *P* < 0.001) was a significant negative predictor, suggesting that increased anxiety was associated with lower self-efficacy. Boredom showed a small but significant positive effect (*B* = 0.604, β = 0.108, *P* = 0.014). Interestingly, boredom exhibited a small but statistically significant positive regression coefficient after adjustment for the other academic emotion dimensions, despite demonstrating a negative bivariate correlation with self-efficacy. This pattern may reflect a suppression effect caused by shared variance among the emotion variables. Therefore, the regression coefficient for boredom should be interpreted cautiously as representing its unique association after accounting for overlapping emotional constructs, rather than a direct positive relationship with self-efficacy. Excitement (*B* = 0.322, β = 0.049, *P* = 0.391) and frustration (*B* = −0.305, β = −0.052, *P* = 0.271) were not significant predictors of self-efficacy.

Multicollinearity diagnostics indicated that all variance inflation factors (VIFs) were below 5, suggesting no significant multicollinearity among the independent variables, as shown in Table 3.

Overall, confidence, satisfaction, and anxiety were the primary academic emotion factors influencing self-efficacy among nursing undergraduates, whereas excitement, frustration, and boredom had relatively small or non-significant effects.

## Discussion

### Relationship between academic emotions and self-efficacy

The present study found that positive academic emotions (e.g., confidence and satisfaction) were significantly positively correlated with self-efficacy, whereas negative emotions (e.g., anxiety and frustration) were negatively correlated. This finding is consistent with the self-efficacy theory proposed by Bandura et al. (2003) which posits that individuals are more likely to develop stronger efficacy beliefs when experiencing positive emotional states, thereby enhancing their confidence in completing academic tasks.

For undergraduate nursing students, whose coursework is highly specialized and practice-oriented, emotional experiences play a particularly critical role in the learning process (Shiu, 1999). Positive academic emotions can enhance learning motivation and engagement, and facilitate the adoption of more proactive and effective coping strategies when facing academic challenges, ultimately promoting higher levels of self-efficacy. In contrast, students who experience persistent negative emotions such as anxiety and frustration are more likely to doubt their own abilities, perceive less control over academic tasks, and consequently exhibit lower self-efficacy.

Furthermore, academic emotions may indirectly influence self-efficacy through their effects on cognitive processing (Wang et al., 2017a). Positive emotions tend to broaden individuals' thought–action repertoires, enhance problem-solving abilities, and improve cognitive flexibility (Wang et al., 2017b), whereas negative emotions may constrain cognitive resources, leading to attentional deficits and reduced learning efficiency (Jiao et al., 2025). Therefore, in undergraduate nursing education, greater attention should be paid to the regulation and intervention of students' academic emotions. Strategies such as optimizing the learning environment, providing psychological support, and implementing emotional regulation training may help foster positive academic emotions, thereby enhancing self-efficacy and ultimately contributing to the development of professional competence and clinical readiness.

### Predictive effects of academic emotions on self-efficacy

The regression analysis indicated that confidence and satisfaction were significant positive predictors of self-efficacy, suggesting that positive academic emotions play a facilitative role in strengthening students' beliefs in their learning capabilities. For undergraduate nursing students, who are required to cope with both intensive theoretical coursework and demanding clinical training, emotions such as confidence and satisfaction not only enhance their perceived control over learning tasks but also increase their intrinsic motivation to engage in academic activities. This, in turn, contributes to the formation of a positive “emotion–cognition–behavior” cycle, which continuously reinforces their level of self-efficacy.

In contrast, anxiety emerged as the only significant negative predictor of self-efficacy, indicating that heightened anxiety may undermine efficacy beliefs through multiple pathways (Ng and Lovibond, 2020; Razavi et al., 2017). Although frustration was negatively correlated with self-efficacy in the bivariate analysis, it did not remain statistically significant in the multivariable model after adjustment for other academic emotion dimensions. This finding suggests that the influence of frustration may overlap with other correlated emotional constructs rather than exerting an independent effect on self-efficacy. On the one hand, anxiety can consume limited cognitive resources, interfere with attention and information processing, and reduce learning efficiency (Basten et al., 2012; Fales et al., 2008). On the other hand, persistent frustration may weaken learning motivation and lead to avoidance of academic tasks, thereby lowering students' evaluations of their own abilities (Ren, 2021). Such a negative cycle is detrimental to the development and maintenance of self-efficacy.

These findings are consistent with the control-value theory proposed by Pekrun (2006), which posits that emotions arise from individuals' cognitive appraisals of control and value, and subsequently influence learning outcomes by affecting cognitive processing, motivation, and the selection of learning strategies. In the present study, confidence and satisfaction may reflect higher perceived control and value, thereby promoting self-efficacy, whereas anxiety and frustration may indicate reduced control or value appraisals, thus exerting inhibitory effects on self-efficacy.

Therefore, greater attention should be paid to the regulation and intervention of academic emotions in undergraduate nursing education. Strategies such as optimizing instructional design, providing appropriate levels of challenge along with timely feedback, and offering psychological support and emotional regulation training may help students effectively manage anxiety and frustration, foster positive academic emotions, and ultimately enhance self-efficacy, contributing to their professional development and clinical competence.

### Interpretation of unexpected regression findings

An unexpected finding was that boredom demonstrated a small positive regression coefficient despite being negatively correlated with self-efficacy at the bivariate level. One possible explanation is a statistical suppression effect resulting from substantial correlations among the academic emotion dimensions. Under such circumstances, regression coefficients represent unique associations after removing shared variance among predictors. Therefore, this result should not be interpreted as evidence that boredom is beneficial for self-efficacy.

Another notable finding was that excitement was strongly correlated with self-efficacy but lost statistical significance in the multivariable model. This pattern suggests that excitement shares considerable variance with confidence and satisfaction, which may explain why it did not contribute additional explanatory power when all emotion dimensions were entered simultaneously.

### Educational implications

Based on the findings of this study, nursing education should adopt a multidimensional approach to systematically enhance students' self-efficacy. First, emotional management education should be strengthened by incorporating emotion regulation training into the undergraduate nursing curriculum. This can help students recognize and regulate negative emotions such as anxiety and frustration, cultivating their tenacious personality traits in the teaching process (Guo et al., 2024), improving their emotional coping skills and providing a solid psychological foundation for the development of self-efficacy. Second, a supportive and facilitative learning environment should be actively created. This includes optimizing classroom climate, promoting interactive engagement between instructors and students, and providing diverse learning resources and practical opportunities. Such an environment can enhance students' learning experiences and satisfaction, thereby increasing their sense of control over learning tasks and their perceived value of academic activities. In addition, the establishment of a positive feedback mechanism is essential. During the teaching process, instructors should provide timely, specific, and constructive feedback to reinforce students' successful experiences, strengthen their confidence, and continuously consolidate and enhance their self-efficacy.

In summary, by combining emotional management education, an optimized learning environment, and effective positive feedback strategies, nursing education can simultaneously influence students' emotions, cognition, and behaviors (Chen et al., 2025). This integrated approach promotes the development of self-efficacy among undergraduate nursing students and lays a solid foundation for their professional growth and clinical competence (Bong et al., 2026; Cho and Kim, 2025).

### Common method bias test

Harman's single-factor test showed that the first factor accounted for 39.057% of the total variance, which was below the threshold of 40%, indicating that common method bias was not a serious concern.

## Limitations

This study has several limitations. First, due to its cross-sectional design, causal relationships between academic emotions and self-efficacy cannot be inferred, and only associations can be identified. Second, the sample was drawn from a single institution, which may limit the representativeness of the findings and constrain their generalizability. Future research could adopt a longitudinal design to explore the dynamic causal relationships between emotions and self-efficacy, and include multi-center or multi-institutional samples to enhance the external validity and broader applicability of the results. Although Harman's single-factor test suggested that common method bias was not a serious issue, this method has limitations and future studies may consider more robust approaches such as CFA marker techniques.

## Conclusion

Academic emotions were significantly associated with self-efficacy among nursing undergraduates. Confidence and satisfaction were identified as independent positive predictors of self-efficacy, whereas anxiety was the only independent negative predictor. These findings suggest that nursing education should strengthen emotional regulation and create supportive learning environments to foster positive academic emotions and enhance students' self-efficacy.

## Data Availability

The data analyzed in this study is subject to the following licenses/restrictions: The datasets used and/or analyzed during the current study are available from the corresponding author on reasonable request. Requests to access these datasets should be directed to Lili Guo, 46469068@qq.com.
